# Looking Forward to the Future of Heparin: New Sources, Developments and Applications

**DOI:** 10.3390/molecules23020293

**Published:** 2018-01-31

**Authors:** Giangiacomo Torri, Giuseppe Cassinelli

**Affiliations:** Carbohydrate Sciences Department of Istituto di Ricerche Chimiche e Biochimiche “G. Ronzoni”, 20133 Milan, Italy; ronzoni@ronzoni.it

The seven reviews and the eleven articles in this special issue provide an updated survey of recent research and developments in the ever-growing field of heparin, along with low molecular weight heparins (LMWHs) and glycosaminoglycans (GAGs). The complex biosynthetic process, and the variability of tissues and animal species, has led to heparin chains heterogeneous in size and both *N*- and *O*-sulfation and *N*-acetylation patterns. Its low concentration in crude extracts, containing other heterogeneous GAGs, leads to a purification process that is very complex, and which is well-guarded by manufacturing companies. Van der Meer et al. [1], through a careful inspection of the academic and patent literature, provide a worthy overview of the multiple steps and variations in purification processes leading to active pharmaceutical ingredient (API)-grade heparin.

As a consequence of the “heparin crisis” in late 2007, an updating of heparin pharmacopeia monographies in the USA and the EU with new NMR and HPLC tests increased the quality control capabilities for crude and API porcine heparins, with some limitations in detecting the addition of non-porcine crude heparins or other GAG-like contaminants. An improvement to this process (Mauri L. et al.) [2] resulted from a collaborative study between the G. Ronzoni Institute and the Division of Pharmaceutical Analysis of Food and Drug Administration (FDA) in the USA. Analyzing 88 samples of commercial crude heparin through an orthogonal approach based on NMR chemometrics along with strong anionic exchange (SAX)—HPLC, they could be differentiated with regard to purity, as well as the mono- and disaccharide composition specific to each GAG family. Furthermore, heparin/heparan sulfate (HS) from different tissues and animal species, as well as from different manufacturing processes, can be characterized, and impurities such as dermatan- and chondroitin-sulfates quantified by the heteronuclear single-quantum correlation (HSQC) NMR approach and multivariate analysis (PCA).

Lima M. et al. [3] reviewed the newer applications of heparins and its analogs, as well as GAGs including marine organisms. The wide range of pharmacological activity of heparin can be attributed to its chemical features, which include heparan sulfate (HS), a widely occurring cell surface-bound polysaccharide, which participates in cell-cell signaling. Most of its potential applications seem to be partially associated with its anti-inflammatory effects, as well as to interactions with a multitude of proteins and inhibition of enzymes involved in pathologic processes, such as heparanase and metalloproteases. Additionally, the role of such mediators as selectins and galectins in cancer and metastasis, cathepsin-d and BACE-1, respectively, in Parkinson’s and Alzheimer’s diseases, human and microbial elastases in cystic fibrosis, and proteases and cytoadherence in parasite infections such as Leishmaniosis are elucidated. The ability to protect from viral infection through enveloped glycoproteins can open other potential applications for heparins and GAGs. 

Two Italian teams (Poli M. et al.) [4] review their studies and recent findings with regard to the role of bone morphogenic proteins (BMPs, members of the TGF-β superfamily heparin/HS binding proteins) in activating the expression of hepcidin, the iron inflammation peptide hormone, which regulates systemic iron hemostasis, and can be deregulated by heparin. An in vivo screening allows the identification of non-anticoagulant glycol-split heparin, delivered by osmotic pumps, and supersulfated LMWH given orally as heparin antagonists and potential candidates for the treatment of anemias in chronic and genetic diseases.

The interactions and binding sites of heparin/HS with BMPs and cytokines of the TGF-β superfamily are reviewed by Rider C. and Mulloy B. [5]. The activity of TGF-β-cytokines in controlling proliferation, differentiation on survival in several cell types are also regulated by a number of secreted BMP antagonist proteins, the majority of which can also bind heparin. In conclusion, potential therapeutic applications of TGF-β cytokines on their own and those with BMP interactions with heparins/HS are described.

In a collaborative study of 6 laboratories in the USA, Europe and India (Bertini S. et al.) [6] the average MW of 20 lots of bovine mucosal heparin (BMH) were determined with the USP monograph method in comparison with porcine mucosal heparin (PMH) and bovine lung heparin (BLH) samples. Even with a wider variation, the average MW of BMH was found to be comparable to that of PMH, while BLH samples had a lower average MW. An alternative method using a polymer-based column with light scattering detection provided results that were in good agreement for all samples investigated in the study. 

An article (Kim H. et al.) [7] reports a study exploring, in different bioreactor conditions, the yield, structure and activity of heparin/HS obtained by expressing serglycin in mammalian cells as an alternative source of these anticoagulant drugs, as well as of new bioengineered analogs.

Three Italian groups (Truzzi E. et al.) [8] explored the possibility of an intestinal lymphatic uptake of an orally formulated heparin. Self-assembled lipid nanoparticles were used to stabilize the heparin-coated iron-oxide nanoparticles. Then, the formulation was characterized with respect to its physical-chemical properties, encapsulation efficacy, in vitro stability, heparin leaking cytotoxicity and indirect indication of lymphatic up-take in CaCo_2_ cells.

A collaborative study by an Israeli and Italian team (Vismara E. et al.) [9] led to the design and identification of a synthetic strategy for obtaining new theranostic super paramagnetic iron-oxide nanoparticle (SPION) systems decorating a magnetic iron-oxide core with an optimized ratio of bioorganic layer and of serum albumin and hyaluronic acid, which was selected to finally include paclitaxel and improve its efficacy. The TD-NMR experiments suggest their suitability for development as contrast agents in MRI.

The review by authors from the Departments of Neurosurgery at two US Universities (Hayman E. et al.) [10] suggests the therapeutic potential of heparins and derivatives for improving outcomes in aneurysm-associated subarachnoid hemorrhage (a-SAH). Retrospective analysis of preliminary clinical studies and experimental works suggest that the pleiotropic effects of heparins can be of benefit in blood-brain barrier dysfunctions, vasospasm, delayed cerebral ischemia and neuroinflammation preventing leukocyte extravasation, modulation of phagocyte activation, and inhibition of oxidative stress, all of which are involved in the complex a-SAH frame.

A Belgian team (Minet V.) [11] reviewed all of the current developed and evaluated functional assays for diagnosis in patients suspected of heparin-induced thrombocytopenia. Drawbacks in some assays, such as platelet activation and Hit antibody detection, are identified as being due to interlaboratory variability, lack of standardization and data control and interpretation.

Compositional analysis of both LMWH Dalteparin (Bisio et al.) [12] and Danaparoid (Gardini C. et al.) [13] and their enzymatically digested oligosaccharides have been determined at the G. Ronzoni Institute, by a combination of the more advanced LC/MS and NMR analytical methods. The API batch-to-batch variability of Dalteparin can also be assessed profiling octa- and deca-saccharides, and fractions endowed with different antithrombin affinity. Chromatographic fractionation and selected enzymatic digestion, as well as NMR analyses of Danaparoid, a GAG complex mixture extracted from porcine intestinal mucosa, allowed the characterization and quantification of the main component as LMW HS, and the minor ones dermatan and chondroitin sulfate, and identified oxidized glucosamine and uronic acid at the reducing ends.

The interactions of Tinzaparin, a LMWH used as antithrombotic prophylaxis in clinical oncology with *cis*-Pt, have been studied “in vitro” and in xenograft models (Mueller T. et al.) [14]. In vitro LMWH can reverse the *cis*-Pt resistance in a cancer-resistant cell line. In vitro preliminary studies show that Tinzaparin has no effect on *cis*-Pt accumulation in *cis*-Pt-resistant xenografts but strongly increases the Pt content in non-*cis*-Pt-resistant ones.

Component fractionation of Semuloparin, an ultra LMWH obtained by a depolymerization process preserving the AT binding region, has allowed a team at Sanofi (Mourier P. et al.) [15] to isolate five octadecasaccharides, each incorporating at least two AT-binding pentasaccharides. Full sequencing and “in vitro” testing of anti-FXa and anti-FIIa activities reveal the peculiarity of the pentasaccharide position within the octadecasaccharides for inhibition potency, which can differ up to twenty-fold in magnitude.

An extended physico-chemical characterization of Fondaparinux, the synthetic α-methyl glycoside of the AT binding pentasaccharide, and the active ingredient of the anticoagulant drug Arixtra^®^, have also been defined on the basis of a determination of single-crystal X-ray conformation. Quantitative NMR were also used, confirming that this method shows intrinsic robustness for content determination (de Wildt et al.) [16].

A team from Heidelberg University (Rappold M. et al.) [17] has synthesized and characterized a more sensitive probe (PDI-1) for the detection of dermatan sulfate by a mix-and-read assay in blood plasma in a clinically relevant concentration range (quantification limit in aqueous matrix 1 ng/mL).

Authors from the Trondheim University (Norway) (Arlov Ø. and Skjåk-Bræk G.) [18] review the synthesis and physico-chemical properties of sulfated alginates used as both a drug delivery system and a biomaterial component. Their superior biocompatibility, mild gelling conditions and structural versatility can open the way for new biomedical applications in fields next to those of GAGs.
